# Telehealth enabled neuropsychological testing (TENT): a new platform for examiner-led, digital cognitive assessment

**DOI:** 10.1007/s00415-026-13732-1

**Published:** 2026-03-09

**Authors:** Chris Tailby, Jodie Chapman, Christoph Helmstaedter, Jonas Haderlein, Graeme Jackson

**Affiliations:** 1 Florey Institute of Neuroscience and Mental Health, Melbourne, VIC Australia; 2 Florey Department of Neuroscience and Mental Health, University of Melbourne, Melbourne, VIC Australia; 3 Department of Clinical Neuropsychology, Austin Health, Melbourne, VIC Australia; 4 Department of Neurology, Bladin-Berkovic Comprehensive Epilepsy Program, Austin Health, Melbourne, VIC Australia; 5 Department of Epileptology, University Hospital Bonn, Bonn, Germany; 6 Department of Medicine (Austin Health), The University of Melbourne, Melbourne, VIC Australia

**Keywords:** Neuropsychology, Cognitive testing, Teleneuropsychology, Telehealth, Epilepsy

## Abstract

**Background:**

Cognitive testing provides an essential marker of brain function. Despite the widespread availability of technology, cognitive testing in contemporary practice largely remains rooted in the manual administration and scoring of analog materials. Here we introduce telehealth enabled neuropsychological testing (TENT): browser-based, videoconference-integrated software for examiner-led cognitive assessment.

**Methods:**

TENT incorporates a battery of tasks assessing memory, language, processing speed, attention and executive functions. We used TENT to conduct remote, telehealth-based assessments in 531 healthy volunteers, and validated the software in a sample of 452 individuals with drug-resistant epilepsy (DRE) and 392 individuals with newly diagnosed seizures. TENT-acquired measures were compared against clinically acquired, in-person, traditional cognitive measures where available. Participant user experience feedback was obtained in a subset of participants.

**Results:**

Comparison of healthy volunteers and DRE participants yielded a pattern of cognitive compromise characteristic of chronic, drug-resistant epilepsy. TENT data was sensitive to demographic and clinical parameters (e.g., age, antiseizure medication load, lateralised structural pathology, age at seizure onset) known to affect aspects of cognition. Correlations between TENT data and reference in-person measures were comparable to published test–retest coefficients for the reference measures. Participant user experience was overall positive.

**Conclusions:**

TENT modernizes traditional neuropsychological testing by providing for human-led cognitive assessments that exploit the benefits of technology-assisted testing and can be used for remote assessment. It offers a modular, normed and standardized system applicable across a range of neuropsychological conditions, providing reach, convenience, efficiencies and data richness. This approach draws upon the strengths of the traditional assessment model while modernizing contemporary neuropsychological practice.

**Supplementary Information:**

The online version contains supplementary material available at 10.1007/s00415-026-13732-1.

## Introduction

Cognitive testing provides an essential marker of brain function. It is used to characterize cognitive functioning in conditions affecting the brain, to track cognition over time, and to monitor the cognitive consequences of interventions. Cognitive testing can provide the first clues as to an emerging pathological process and conversely can demonstrate remarkably preserved cognitive functioning in the face of substantial structural and functional brain compromise [1].

Contemporary cognitive testing approaches can be divided into two main classes [2, 3]: supervised, examiner-led testing and automated, unsupervised, computer-based testing. *Supervised, examiner-led cognitive testing* is the most common approach. Traditionally, the assessment is conducted in person using analog materials. Responses are typically recorded, scored, and normed manually; then where necessary, manually transcribed into a central database. This style of practice has remained largely unchanged for over 100 years, in many cases using cognitive tests that have themselves changed very little over this period [4].

More recently, this in-person approach has been adapted for videoconference telehealth [5], by ‘shoehorning’ existing practices into the videoconference medium with the aim of faithfully reproducing traditional assessments. In the move to teleneuropsychology, little to no attempt has been made to exploit the opportunities afforded by the technology in use [3]: stimuli are delivered via screen share and/or document camera (rather than embedded in the testing interface), with responses recorded and scored manually on standard paper forms^1^.

With *automated, unsupervised, computer-based cognitive testing* the assessment is computer administered, without the oversight of a human examiner [2, 3, 6, 7]. Tasks are introduced and explained via a mix of text, audio and video. Participant responses are recorded directly through the software, via keyboard, mouse and/or touchscreen, which also enables collection of response timing information (e.g., reaction times). Scoring and normative conversion is automatic, and results are stored in electronic form. Such assessments can, in principle, be completed anywhere and at any time via an internet-connected device. This greatly increases both the reach and ease of participation (e.g., no need for travel to and from a clinical/research facility), and scalability. While these features are attractive, elimination of a human examiner leads to notable downsides [3, 7], including the inability to detect and manage participant misunderstanding and participant disengagement, both of which lead to high rates of invalid data, and failure to sample key domains (e.g., recollection, as opposed to just recognition).

Here, we introduce Telehealth Enabled Neuropsychological Testing (TENT) [3], software that enables a new approach to neuropsychological assessment combining the benefits of examiner-led assessment, the extensive evidence base underpinning traditional pen-and-paper based cognitive testing, the advantages of computer-assisted assessment, and the reach of videoconference-based testing. We describe the development and application of the TENT software. We then illustrate the utility of TENT for delivering browser-based remote videoconference-based assessments. We do this by demonstrating TENT’s *criterion validity* by (1) comparing performance on TENT tasks in a large sample of individuals with drug-resistant focal epilepsy (*n* = 452) against that of a large sample of healthy volunteers (*n* = 531), and (2) evaluating the sensitivity of TENT tasks to demographic and clinical parameters (e.g., age, antiseizure medication load, lateralised structural pathology, age at seizure onset) known to affect aspects of cognition. We demonstrate the *convergent validity* of TENT tasks by comparing TENT-acquired data against data acquired from in-person, traditional cognitive measures. We demonstrate the *usability* of TENT by evaluating rates of data validity and the effect of examiner experience on cognitive scores derived from TENT. We finally demonstrate the *acceptability* of TENT by evaluating participant evaluations of their experience in TENT assessments.

## Methods

### Participants

Participants are adults aged between 18 and 67 years recruited to the Australian Epilepsy Project [AEP; epilepsyproject.org.au; 8]. The AEP recruits individuals with drug-resistant focal epilepsy (DRE), with newly diagnosed epilepsy (< 6 months; NDE), or with a first unprovoked seizure (FUS), as well as healthy volunteers. All participants are required to have a functional level of English; exclusion criteria are a moderate or severe intellectual disability and/or contraindications for 3 T MRI. Additionally, healthy volunteers cannot have a history of seizures or epilepsy, a history of other neurological conditions (e.g., acquired brain injury, neurodegenerative disorder), and/or an active severe or progressive illness. Individuals with a history of seizures (DRE, NDE, and FUS) were referred by neurologists in private or public health services across Australia. Healthy volunteers were recruited via word of mouth, advertisements and social media.

The main analyses compared TENT cognitive scores in the DRE (*n* = 452) and healthy volunteer groups (*n* = 531; Figs. 2 and 3A, B). Secondarily, we examined the sensitivity of certain TENT measures to antiseizure medications (ASMs), age, unilateral structural damage, and age of epilepsy onset; and examined convergent validity by comparing TENT scores to scores on traditional in-person measures (see below; Fig. 3C, D and Table 3). To achieve these secondary aims, we incorporated additional data from people newly diagnosed with seizures (NDE = 287; FUS = 105).

### Software design and implementation

**Fig. 1:**
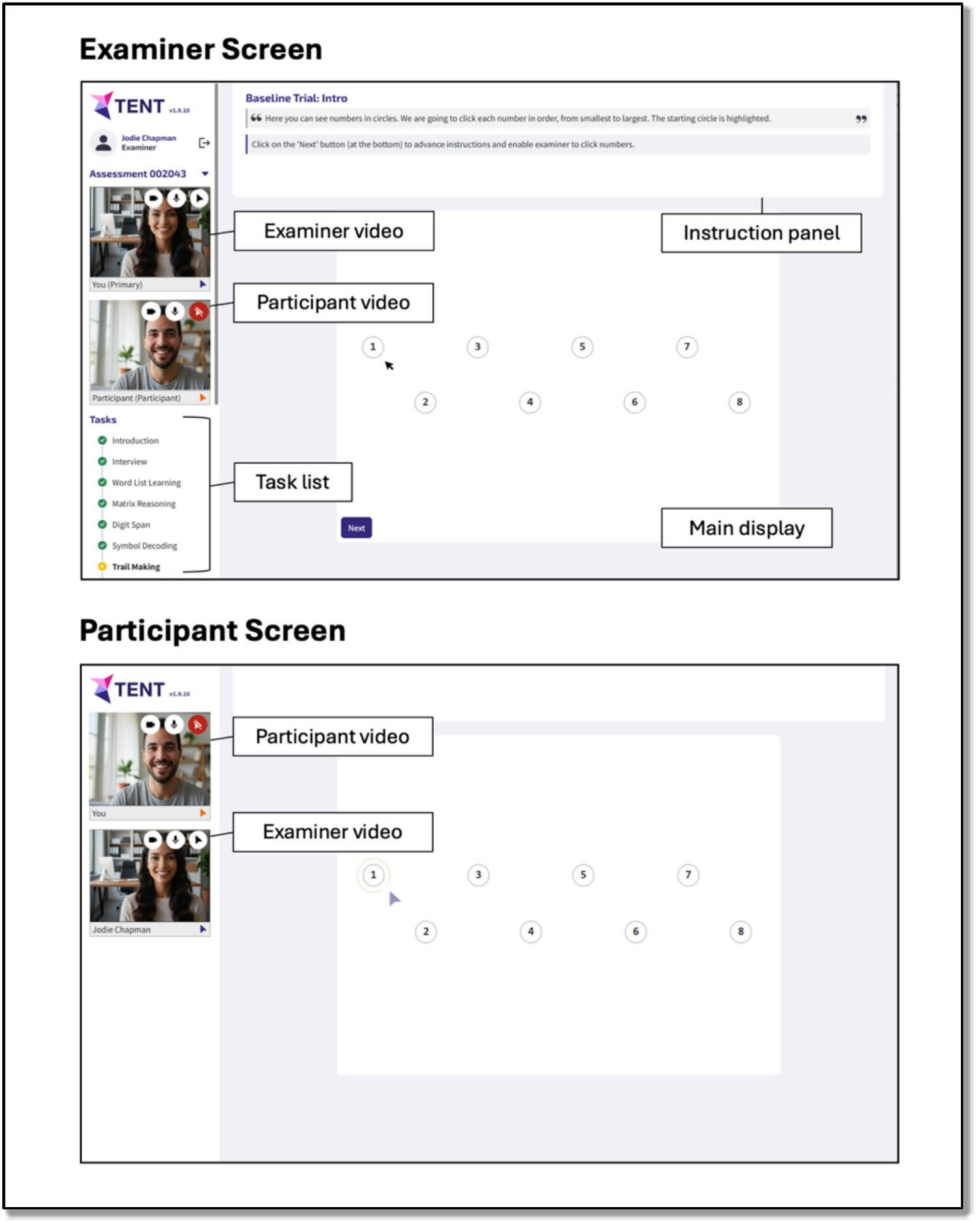
Basic layout of the TENT software, showing examiner screen (top) and participant screen (bottom)

TENT is custom-built videoconference-integrated browser-based software, compatible with a camera and microphone enabled desktop/laptop (with a connected mouse), or tablet device with a connected mouse/keyboard. The cognitive tasks described below are administered via the software, and responses are recorded therein. The layout is illustrated in Fig. 1. Video feeds of the participant and examiner are visible to both parties throughout the entire session as tiles at the left of their screens. The participant additionally sees the examiner’s video feed on the main display when the task allows (i.e., when no stimulus material is displayed).

Examiner and participant screens differ. The examiner screen shows:

1. On the left, an ordered task list, including the status of each task (i.e., completed, in progress, yet to commence). In the AEP, tasks are administered in a predefined order, but it is possible to navigate through the tasks flexibly if required, including omitting tasks where necessary (e.g., participant refusal).
2. At the top, explicit task instructions. These include instructions the examiner is to read aloud verbatim to the participant and prompts for the examiner that facilitate task administration and scoring.
3. On the main display, a real-time mirror of the participant’s screen and cursor to facilitate observation of task performance, and/or response buttons and text fields that the examiner can use to record participant responses.

The participant main display shows either a full screen video feed of the examiner or task stimuli. Depending on the task, participants respond either via mouse clicks or their keyboard or verbally with their responses recorded by the examiner via the examiner interface. Sessions were not audio/video recorded (though this could readily be achieved with appropriate ethics approval and participant consent).

TENT includes a screenshare feature, enabling the administration of cognitive tasks not built into the software.

### Measures

TENT tasks were selected and adapted from measures with an existing evidence base in the neuropsychological literature broadly, measures with demonstrated utility across the different stages of adult epilepsy (e.g., new onset and chronic), recommendations from international epilepsy organizations (e.g., NINDS Common Data Elements for Epilepsy [9]; EpiCARE European Reference Network [10], International League Against Epilepsy [11]), and to ensure coverage across cognitive domains commonly affected in epilepsy. Tasks were adapted or developed de novo for administration via TENT, drawing upon key principles and attributes underpinning conventional in-person materials with demonstrated utility. A summary of each task is provided in Table 1 (with more detail available in Supplementary Information).

**Table 1:** Cognitive tasks implemented in the TENT software (alphabetical)

Task	Description	Primary measure (s)	Major domain (s)
Category Fluency	Name as many items as possible belonging to a given category	Total valid words produced within 60s	Language
Confrontation Naming	Name photographs of objects	Total items correctly named	Language
Digit Span	Accurately repeat, either forwards or in reverse, strings of numbers of increasing length	Longest span forwards Longest span backwards	Attention Working memory
Figural Learning	Learn and reproduce from memory eight visual figures imposed upon an underlying grid	Total items recalled across three learning trials	Nonverbal recall memory
Figural Recognition	Identify from among distractors the stimuli seen during figural learning	Total correct configuration and position recognition	Nonverbal recognition memory
Finger Tapping	Tap a key on the keyboard as rapidly as possible, for 10 s, using the index finger of each hand	Mean tap rate with the dominant hand Mean tap rate with the non-dominant hand	Motor function
Irregular Word Reading	Pronounce irregular words	Total number of correctly pronounced words	Language Intellect
Letter Fluency	Name as many words as possible beginning with a particular letter	Total number of valid words produced across three separate letter trials, each lasting 60s	Language Executive function
Reaction Time	Respond, by keypress, as soon as a green circle appears on the screen	Median reaction time	Simple reaction time
Spatial Stroop	Respond by keypress, using either the left or right index finger, as quickly as possible following presentation of the words LEFT or RIGHT on the screen	Median congruent reaction time (i.e., LEFT = respond left) Median incongruent reaction time (i.e., LEFT = respond right)	Choice reaction time Attentional control
Symbol Decoding	Orally transcribe symbols into their corresponding numbers based on a key	Total correct oral transcriptions achieved within 90s	Processing speed
Trail Making	Trace out via mouse clicks a path connecting randomly arranged numbers in ascending order (Part A), or randomly arranged numbers and letters in alternating ascending order (Part B)	Total completion time for part A Total completion time for part B	Processing speed Divided attention
Word List Learning	Spontaneous recall of a list of 15 unrelated words presented across three learning trials	Total words recalled across the three learning trials	Verbal learning
Word List Delay	Spontaneous delayed (15 + mins) recall of words from word list learning	Total words recalled on delay	Verbal delayed recall
Nonword Rhyming*	Determine if two non-words would rhyme if pronounced aloud	Total number of accurate rhyme judgements	Language
Spatial n-Back*	Stimuli are dots shown briefly (0.75s; 1.25s ISI) at one of four possible locations. Participants indicate, via button press, either where the current dot is shown (0-back) or where the previous dot was shown (1-back)	Proportion correct (0-back) Median reaction time (0-back) Proportion correct (1-back)	Choice reaction time Attention Working memory

*Indicates tasks used principally for task fMRI training

#### *Additional cognitive tasks delivered *via* screen share*

Intelligence was measured using the full-scale IQ (FSIQ-2) index from the Wechsler Abbreviated Scale of Intelligence Second Edition (WASI-II) [12], calculated from scores on the Vocabulary and Matrix Reasoning subtests. These measures were administered using the screenshare feature within the TENT software, with stimuli accessed via Q-Global, and responses recorded manually.

### Procedure

This research was completed in accordance with the Helsinki Declaration. Ethics approval for the AEP was granted by Austin Health Human Research Ethics Committee (HREC/68372/Austin-2022). Participants provided their informed consent prior to participation. Demographic and clinical data were obtained during eligibility screening, from the participants’ treating clinician, and/or from a medical history interview conducted as part of the AEP.

Trained research assistants conducted the cognitive testing sessions. Research assistants were either individuals with postgraduate training in clinical neuropsychology, or individuals with undergraduate training in psychology or experience working in clinical trials. Training involved (sequentially): (1) observation of a training examiner conducting a session, (2) a general introduction to TENT, including the rationale and methods underlying each task and a Standard Operating Procedures manual, (3) internal practice (mock) assessment(s) of colleagues, (4) an observed mock assessment(s), (5) an observed assessment(s) with a healthy volunteer, and (6) an observed assessment(s) with a participant with epilepsy, ultimately used to assess readiness to undertake independent data collection. Trained examiners are reobserved yearly to ensure compliance with standardized assessment procedures.

Participants completed assessment sessions either at home on their own device (*n* = 1282, 93.2%), or at a research site on a provided device while the research assistant conducted the session in a separate room (*n* = 93, 6.8%). The latter was the case when participants did not have an appropriate device or internet connection, or for other reasons were unable to complete the session at home. The TENT software automatically captured details of the participants’ browser (name, version, window size), operating system (name, version), and monitor resolution. In each session, the research assistant went through a checklist with participants to ensure the environment in which they were conducting the session was appropriate. This included, for example, ensuring they were alone in a private, distraction-free environment, that computer notifications were turned off, and confirming their location, phone number, and emergency contacts in the event of a seizure. For all neuropsychological measures, the administering research assistant characterized the completion of the test as ‘complete and reliable’, or impacted by other factors (e.g., distraction, refusal). Only data marked as ‘complete and reliable’ were included in analyses. During each task the examiner was also able to type in comments, embedded in the electronic data file, to facilitate the later interpretation of performances.

Follow-up online surveys about the participants’ experience using the TENT software were collected in a subset of participants (*n* = 262 healthy volunteers, 251 DRE, 165 NDE and 63 FUS), as these were introduced later during data collection. Specifically, participants were asked, “How would you rate the online neuropsychology experience, between 1 and 5?” on a scale from 1 (*Did not enjoy)* to (*Excellent*). A free text field was also available for qualitative feedback.

### Assessment of convergent validity

A subset of participants also underwent in-person clinical neuropsychological assessment as part of their routine clinical care. For these participants, we were able to compare performances (where available, and without a neurosurgical procedure occurring between assessments) on standard cognitive tasks administered during these clinical assessments with TENT measures assaying similar cognitive constructs (i.e., convergent validity). Specifically, we compared: scores on TENT *Word List Learning* and *Word List Delay* with scores on a clinically acquired Rey Auditory Verbal Learning Task (RAVLT; [13, 14]; median interval between tests = 80.5 days; IQR = 174.8 days; AEP preceded clinical in 41 of 86 cases); scores on TENT *Figural Learning* with delayed (10 + min) recall scores on the Rey Complex Figure Test (RCF) [15]; median interval between tests = 73 days; IQR = 192.5 days; AEP preceded clinical in 24 of 43 cases); and scores on TENT *Confrontation Naming* with scores on the Boston Naming Test (BNT) [16]; median interval between tests = 76.5 days; IQR = 137.8 days; AEP preceded clinical in 24 of 40 cases). We have also reported elsewhere on the convergent validity of scores on the *Symbol Decoding* and *Trail Making* tasks relative to their traditional pen-and-paper in-person formats [17]. Convergent validity of reaction time tasks was assessed by comparing reaction times on the *Spatial n-Back* task in TENT to the reaction times on the same measure during the subsequent AEP MRI scan (as measured via a Cedrus high temporal precision button box; median interval between tests = 9 days; IQR = 13.8 days).

### Statistical analyses

Raw scores on metrics derived from TENT tasks were converted to *z*-scores by fitting a series of Bayesian hierarchical regression models (to be described in detail elsewhere). In brief, for each test metric, all AEP participants’ (those with a history of seizures and healthy volunteers) raw scores were fit simultaneously in a single model that estimated the overall effects of age (in years), level of education (in years), and assigned sex (categorical), plus group specific (categorical: history of seizures, healthy volunteer) deviances from these overall effects, separate intercepts for group (history of seizures, healthy volunteer), and an offset for English not as a primary language status, where indicated (categorical: English first language, English not first language). For each test metric, underlying empirical data distributions were fit using (truncated) Gaussian, Gamma, or negative Binomial distributions as appropriate.

The hierarchical Bayesian regression model for each test metric was fit using partial pooling (i.e., the parameter estimates for the seizure history and healthy volunteer samples were informed and constrained by one another). We used python version 3.12 and the Bayesian modeling framework *pymc*. From these models, an individual’s raw score can be expressed as a *z*-score capturing its deviation from the model predicted score for any of the observed groups. Thus, models effectively express each raw score as a standardized deviation from a predicted score based on (adjusted for) age, assigned sex, level of education, English not as a primary language status, and group membership. In the following, we report all scores as z-scores relative to the healthy volunteer group.

Further downstream analyses were conducted in R version 4.4.2 within R Studio version 2024.12.1 + 563. The *criterion validity* of TENT measures was assessed by comparing *z*-scores in the DRE group with healthy volunteers, and by evaluating whether demographic or clinical factors (e.g., age, ASM load) affected performance, each done by using boxplots and scatterplots for visualization. Sensitivity to the laterality of mesial temporal lesions was assessed by comparing, via MANOVA, memory and naming measures (Word List Learning, Word List Delay, Figural Learning, Confrontation Naming) in patients with either unilateral hippocampal sclerosis (HS) or prior anterior temporal lobectomy (ATL). Sensitivity to worse cognitive outcomes in those with earlier epilepsy onset was evaluated by running a principal components analysis (PCA) on the metrics derived from the TENT-integrated tasks (excluding WASI-II measures) and correlating scores on the first principal component against age at seizure onset. *Convergent validity* was assessed by calculating the Pearson correlation between TENT scores and scores obtained during in-person assessments. The convergent validity of reaction times were assessed by calculating the intra-class correlation coefficient (ICC) between (log transformed) TENT derived reaction times and Cedrus response box derived reaction times. ICCs were estimated using the ICC function from the “psych” package version 2.5.3 (type = ICC3, a two-way mixed effects model assessing for consistency [18]). To evaluate the *usability* of TENT, comparison of participant scores obtained from assessments administered by different groups of examiners (those with and without postgraduate neuropsychology training) were compared using Bayesian *t*-tests, with Bayes factors (obtained via the BayesFactor package, version 0.9.12.4.7, in R) used to evaluate evidence in favor of the null hypothesis of no difference between examiners, and Bayesian equivalence tests [using the ttestBF function in the BayesFactor package and specifying nullInterval = c(− 0.1,0.1)] to evaluate evidence that any observed differences between examiner groups fell in the range −0.1 to 0.1. We also evaluated the rates of valid data on TENT Tasks. *Acceptability* was evaluated by looking at the quality ratings and participant qualitative feedback.

## Results

### Participant demographics

Participant demographic information is reported in Table 2. Regarding the main analysis, healthy volunteer and DRE groups were comparable in age; however, the healthy volunteer sample contained a larger proportion of females, had completed more years of education, and had a higher FSIQ-II. Within the DRE group, 52% of cases had temporal lobe epilepsy, and the group as a whole was taking a median of three ASMs at the time of testing.

**Table 2:** Participant demographics

	Participants in main analyses comparing healthy volunteers and DRE		Additional participants included in analyses of ASM effects and convergent validity
	Healthy volunteer (*n* = 531)	Drug resistant epilepsy (*n* = 452)	Newly diagnosed seizure (s) (*n* = 392)
Sex			
Female	363 (68.4%)	235 (52.0%)	208 (53.1%)
Male	168 (31.6%)	217 (48.0%)	184 (46.9%)
Age			
Mean (SD)	38.2 (14.0)	36.3 (12.1)	36.0 (14.0)
Years of Edu			
Mean (SD)	14.8 (2.3)	12.8 (2.25)^1^	13.3 (2.45)
FSIQ			
Mean (SD)	110 (12.3)^2^	95.2 (14.5)^3^	101 (13.8)^4^
Age of onset			
Mean (SD)	–	19.1 (12.7)^5^	32.6 (14.6)^6^
Localization			
Extratemporal	–	130 (28.8%)	24 (6.1%)
Temporal	–	236 (52.2%)	107 (27.3%)
Unknown	–	86 (19.0%)	261 (66.6%)
Num ASMs			
Median [Min, max]	–	3 [0,5]	1 [0,3]

^1^Two cases with missing data

^2^23 cases with invalid data

^3^31 cases with invalid data

^4^17 cases with invalid data

^5^21 cases with missing data

^6^5 cases with missing data

### DRE vs healthy volunteers on main measures from TENT

**Fig. 2:**
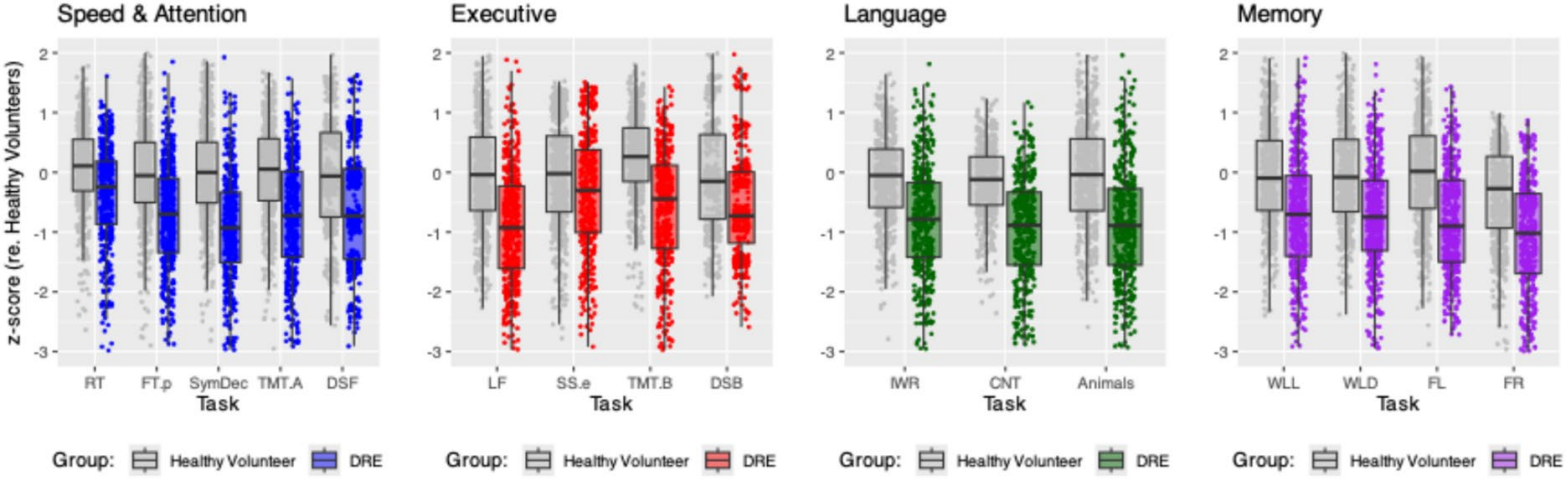
Age, sex and education adjusted z-scores for healthy volunteer (grey boxplots) and DRE (colored boxplots) groups on selected TENT cognitive measures. Cognitive tasks have been categorized according to the principal cognitive domain assessed. *RT* = Simple Reaction Time. *FT.p* = Finger Tapping with preferred hand. *SymDec* = Symbol Decoding. *TMT.A* = Digital Trail Making Test Part A. *DSF* = Digit Span Forward. *LF* = Letter Fluency. *SS.e* = Spatial Stroop errors. *TMT.B* = Digital Trail Making Test Part B. *DSB* = Digit Span Backward. *IWR* = Irregular Word Reading. *CNT* = Confrontation Naming Test. *Animals* = Animal Fluency. *WLL* = Word List Learning. *WLD* = Word List Delay. *FL* = Figural Learning. *FR* = Figural Recognition

Demonstrating the criterion validity of TENT tasks, participants with DRE exhibit the expected pattern of reductions in *processing speed/attention*, *executive function*, *language*, and *anterograde memory* relative to healthy volunteers (Fig. 2).

### Validation against demographic and clinical variables

**Fig. 3:**
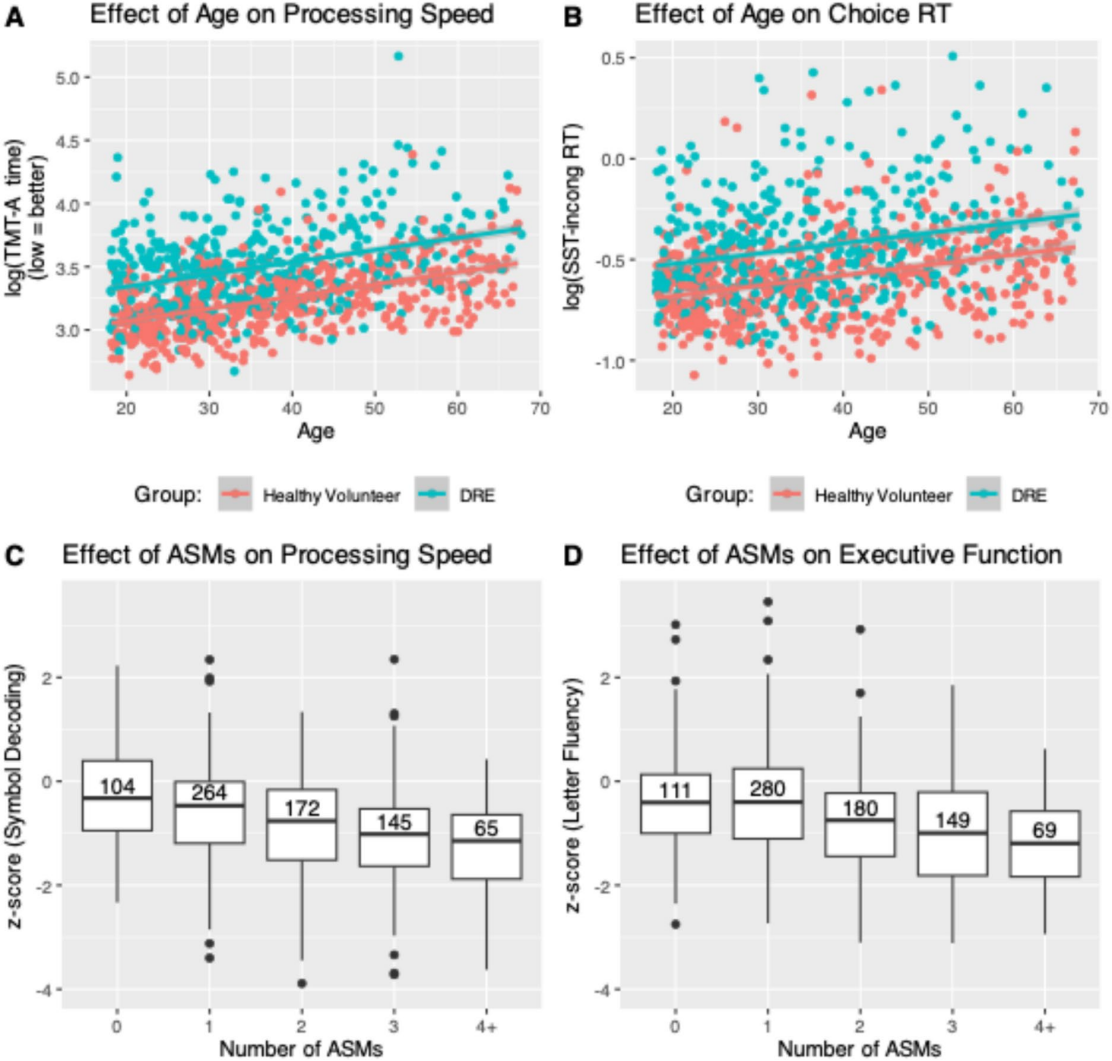
TENT data reproduces the expected detrimental effects of age (**A**, **B**) and antiseizure medication load (**C**, **D**) on aspects of cognition. Processing speed (**A**) and choice reaction times are slowed with increasing age. Processing speed (**C**) and letter-based verbal fluency (**D**) are reduced with increasing numbers of antiseizure medications taken (number of participants contributing to each boxplot is reported within each boxplot)

To further establish the criterion validity of TENT tasks we examined the effects of demographic and clinical variables known to be associated with variability in cognitive performance. As expected, we observed age-related declines in information processing speed and choice reaction times via the TENT software (Fig. 3A, B). Similarly, we observed a detrimental effect of increasing ASM load on processing speed and executive function (Fig. 3C, D).

We tested for lateralised effects by comparing the performance of cases with left medial temporal lesions (*n* = 35; comprising 26 with left HS and 9 with left ATL) against those with right medial temporal lesions (*n* = 19; 14 with right HS and 5 with right ATL). A MANOVA including Word List Learning, Word List Delay, Confrontation Naming and Figural Learning as dependent variables, and FSIQ-II as a covariate, was significant (*F*(4,48) = 6.7, *p* < 0.001) with a large effect size (*η*^2^ = 0.36). Consistent with known laterality effects in mesial temporal lobe epilepsy [19–22], this was driven by lower scores in the left medial temporal lesion group on Word List Delay (*F*(1,51) = 11.4, *p* = 0.002, *η*^2^ = 0.17) and Confrontation Naming (*F*(1,51) = 3.7, *p* = 0.030, *η*^2^ = 0.09).

And lastly, we tested for the expected pattern of worse overall performance in cases with earlier epilepsy onset [23–26] by running a PCA on metrics from all TENT integrated tasks (i.e., excluding WASI-II measures), then correlating scores on the first principal component (explaining 33% of the total variance) against age of onset. This yielded a significant correlation (*r*(568) = 0.20, *p* < 0.001, 95% CI = [0.11, 0.27]), indicating worse overall cognitive performance in those with an earlier age of seizure onset. By comparison, the correlation between IQ and age of onset was slightly weaker (*r*(568) = 0.15, *p* < 0.001, 95% CI [0.07, 0.23]).

### Validation against reference measures

Table 3 reports correlations between raw scores on selected TENT tasks against raw scores on standard neuropsychological tasks collected in person either during their site visit for MRI scanning (Written Symbol Digit Modalities Task [SDMT], TMT-A and TMT-B; see [17]) or during routine in-person clinical assessments. Correlations range between 0.61 (digital versus written TMT-A) and 0.93 (Confrontation Naming versus BNT). We note that the observed correlations are on the order of the published test–retest reliability of the reference instruments themselves (see Table 3), indicating moderate to strong concurrent validity.

**Table 3:** Validation of selected TENT tasks against reference measures

TENT task	Reference task	*n*	*r *(TENT vs ref)	Test–retest of reference task
Symbol Decoding	Written SDMT	128	0.76*	0.70–0.91^a^
TMT-A	Written TMT-A	162	0.61*	0.46–0.79^b^
TMT-B	Written TMT-B	156	0.71*	0.44–0.89^b^
Word List Learning score	RAVLT: sum(A1-A5)	86	0.71	0.60–0.70^c^
Word List Delay score	RAVLT: delay	81	0.63	0.60–0.70^c^
Figural Learning	RCF: delay	43	0.60	0.59–0.79^d^
Confrontation Naming	BNT	40	0.93	0.62–0.94^e^
0-back 4AFC RT (Reaction Time)	0-back 4FC RT (Cedrus)	674	0.70	0.54–0.81^f^

*Convergent validity for Symbol Decoding and Trail Making tasks as reported in [17]

^a^References for test–retest for SDMT [27–30, 30]

^b^References for test–retest for TMT [31–34]

^c^References for test–retest for RAVLT [35–37]

^d^References for test–retest for RCF [33, 35, 38]

^e^References for test–retest for BNT [39–41]

^f^References for test–retest for RT [42–46]

We validated the Irregular Word Reading task against WASI-II FSIQ-2. The observed correlation of 0.70 (*r*(900) = 0.70, *p* < 0.001, 95% CI [0.67, 0.73]) compares favorably with National Adult Reading Test-IQ and Test of Premorbid Functioning-IQ correlations reported in the literature (0.55–0.74 [47–50]).

There was a strong relationship between choice reaction times measured via the TENT software and those measured on a high temporal precision Cedrus response box (*r* (672) = 0.70, *p* < 0.001, 95% CI [0.66, 0.73]; ICC = 0.69, 95% CI [0.65, 0.73]). This compares favorably with the ICCs reported for dedicated reaction time testing systems [42–46].

We do not have matched reference data for the TENT Finger Tapping test. We observed tapping rates of 59.4 ± 9.6 (µ ± SD) for the preferred hand and 54.8 ± 7.9 for the non-preferred hand in healthy volunteers. This is comparable to the data of Hubel et al. [51], who reported tapping rates of 56.3 ± 9.6 and 50.4 ± 8.5 for the preferred and non-preferred hands, respectively, using a computer-administered tapping task in a sample of 1519 healthy adult participants. Their slightly lower rates may reflect the slightly older mean age of their sample (45.8 years versus 37.6 years in our sample).

### Comparable data obtained by examiners with and without postgraduate training in neuropsychology

Our team of examiners included individuals with (*n* = 8) and without (*n* = 10) postgraduate clinical neuropsychology training. To assess whether this difference in examiner training affected the obtained data, we compared scores on TENT-integrated cognitive tasks from assessments of healthy volunteers and individuals with DRE administered by examiners with (*n* = 458 assessments) and without (*n* = 525 assessments) postgraduate clinical neuropsychology training. For all tasks, Bayes factors (BF_01_) indicated moderate or greater support for the null hypothesis of no difference between examiner groups, ranging from BF_01_ = 4.1 for Word List Learning to BF_01_ > 13 for Word List Delay and Figural Recognition. Bayesian equivalence testing also indicated strong or greater evidence that the difference between examiner groups fell within the range −0.1 to 0.1, with Bayes factors ranging from 10.0 for Word List Learning to > 60 for Word List Delay and Figural Recognition.

### Positive user experience with TENT

Of 22,000 cognitive tasks administered and scored in TENT (16 tasks in each of 1375 participants: 531 healthy volunteers, 452 individuals with DRE and 392 individuals with newly diagnosed seizures), 21,373 (97.2%) were marked as ‘complete and reliable’. Of the 627 tasks not marked as ‘complete and reliable’, 229 were marked as ‘technical issue’ (across 177 participants). The remainder were invalid for reasons other than the technology (i.e., 132 in which English not as a primary language was flagged as impacting performance, across 66 participants; 108 in which instructions were not understood or followed correctly, across 90 participants; 62 due to distraction, across 53 participants; 15 due to refusal, across 11 participants; 11 due to seizures, across 8 participants; the remainder were marked as ‘other’).

Participant user experience feedback obtained from 741 participants (262 healthy volunteers, 251 individuals with DRE, 165 individuals with NDE and 63 individuals with FUS) indicated that 89% of participants rated their online cognitive testing experience using the TENT software as 4 out of 5 or greater (median = 5, interquartile range = 1). Positive qualitative feedback emphasized the convenience and scope of participation from home, and ease of use of TENT, e.g.: “The neuropsychology testing was excellent from a technological standpoint and I much preferred it to the manual one with the blocks etc. that I performed at the [*hospital*] before my video EEG monitoring. Interaction with it was intuitive and I felt less anxious with the process”; “Very convenient. I was wondering if I wouldn’t be as engaged because I wasn’t face to face, but it was very interactive and user friendly which I think helped a lot”; “It made it far easier to find a time to do the video call—ultimately it meant there was more flexibility which is always great! It also meant I was in a comfortable environment”; “It was much more efficient. I couldn’t have participated if I had to be in [*the city*]”. Negative qualitative feedback generally related to technology issues relating to personal equipment, e.g.: “[I would have liked the] ability to test my laptop prior. First some reason my camera and mouse were misbehaving.”

## Discussion

We developed and validated novel cognitive assessment software, named Telehealth Enabled Neuropsychological Testing (TENT). TENT is videoconference-integrated, browser-based cognitive testing software allowing for human-led, technology-embedded, rich cognitive characterization administered remotely or in person. We have implemented a suite of cognitive tasks in TENT assaying a broad spectrum of cognitive domains central to neuropsychological assessment across a range of conditions. We have presented data obtained remotely from over 500 healthy volunteers, 400 individuals with DRE and over 300 people with newly diagnosed seizures, demonstrating the sensitivity of the testing approach to relevant demographic and clinical factors, and validity with respect to in-person traditional assessments.

### TENT is sensitive to demographic and clinical variables

The literature on drug-resistant focal epilepsy, based on in-person traditional cognitive assessments, has consistently shown reductions in processing speed, attention/working memory, executive functions, language, and anterograde memory relative to healthy volunteers [52, 53]. TENT assessment reproduces this expected pattern of findings demonstrating the sensitivity and validity of the software (Fig. 2). TENT assessment also captured the expected pattern accompanying left versus right unilateral mesial temporal damage [19–22], and the deleterious effects of earlier age of seizure onset on overall cognitive ability [23–26].

The data reported here also shows that TENT scores were affected by variables widely documented to influence performance in specific cognitive domains. For instance, as expected, performances on TENT measures of information processing speed and reaction time declined with increasing age [54], for both the healthy volunteer and the DRE groups (Fig. 3). Similarly, as expected, higher ASM load was associated with poorer performances on measures of information processing speed and executive function (Fig. 3). Traditional, in-person assessments have shown that speed and executive functioning are among the domains most sensitive to ASM effects [55, 56]. The sensitivity of TENT to ASM effects is potentially of important clinical utility, as the software could be used to conduct brief, remote cognitive screening of patients in the period following medication adjustments. Doing so would provide a simple, practical and objective assay for adverse cognitive side effects without requiring in-person clinic visits. This could significantly improve the feasibility, tolerability and reach of routine neuropsychological follow up and outcome monitoring, not just with regards to ASM changes but also following interventions such as neuromodulation and surgery, and more broadly outside the context of epilepsy care.

### TENT-based measures demonstrate convergent validity

The data presented here (Table 3) validate a novel photo-based Confrontation Naming task implemented in TENT against arguably the ‘gold standard’ confrontation naming task, the BNT [9, 57, 58]. In future work we plan to further refine and extend this instrument, by eliminating any problematic items (e.g., those with strong racial or cultural biases [59]) and deriving normative metrics for latencies. Current measures of confrontation naming define performance primarily based on accuracy, regardless of latency [though see 60, 61]. Such accuracy-based tasks would fail to detect changes in individuals whose accuracy scores remain stable over time (e.g., pre- vs post-surgery), despite changes in the efficiency of word retrieval.

For our measure of verbal learning we adapted the five trial RAVLT, using a novel word list administered over three learning trials and a delayed recall trial (i.e., omitting also the interference list from the RAVLT). This provides a briefer overall administration time, while still tapping into verbal learning and delayed recall. Here we provide strong evidence of convergent validity between TENT Word List Learning and Delay and the RAVLT. The same holds for our convergent validation of TENT Figural Learning (Table 3). Importantly, the TENT Figural Learning task simultaneously solves the challenge of assessing non-verbal memory remotely and making scoring objective. Most measures of ‘non-verbal’ recall (e.g., RCF [15]; Visual Reproduction from the Weschler Memory Scale – Fourth Edition [62]; Brief Visuospatial Memory Test [63]; DCS [20]) require examiner judgment about the ‘adequacy’ of a reproduction, introducing subjectivity into scoring. Scoring of the TENT Figural Learning task is entirely objective. Furthermore, storing individual item-level data affords the opportunity (applicable to other TENT tasks also) to apply machine learning approaches to identify error patterns associated with specific patterns of pathology (e.g., right versus left temporal damage; frontal vs non-frontal damage). Such error pattern analysis has long been of interest in neuropsychology [e.g., 64, 65].

We did not collect concurrent validation data for the TENT Finger Tapping task. Despite this, as noted in the Results, our healthy volunteer data is comparable to that reported by Hubel et al. [51] who also used a computer-based tapping procedure. We note that our and Hubel et al.’s [51] mean tapping rates are slightly higher than those obtained with traditional tapping devices (e.g., Halstead–Reitan), consistent with evidence that electronic keyboards or mice—requiring less force and depth of movement—produce rates that are higher but strongly correlated with traditional instruments [66].

The validity of the videoconference administration of other tasks administered in TENT has already been independently demonstrated. This includes measures of letter- and category-based verbal fluency, digit span, reading of irregularly spelled words, and word list learning tasks [for a recent review and meta-analysis, see 67]. We have also recently reported on the convergent validity of the Symbol Decoding and Trail Making tasks implemented in TENT [17].

### Positive user experience with TENT

Our data indicate a positive participant and examiner user experience with TENT. Participant comments indicated that they appreciated the ability to conduct testing from their own home, eliminating the need to travel to and from a research facility, and found the software easy to use. On the examiner end, the high rate of usable data (~ 97%) attests to the reliability of the software even when administered remotely. This high rate of usable data compares favorably against fully automated, computerized remote testing methods which we have previously shown can result in data invalidation rates of ~ 10%, largely secondary to participant disengagement and misunderstanding [3]. The use of a human examiner in TENT is an important element in detecting and managing such issues.

This positive feedback from participants was evident even given the technological considerations involved in using TENT. TENT requires that participants have access to an appropriate device and internet connection. A vast majority of AEP participants (93.2%) completed the assessment from their home on their own device. Further, while TENT does not require participants to download and install bespoke software onto their device, there was occasionally some troubleshooting required (primarily around browser permissions relating to microphone and camera) to ensure the videoconferencing features worked appropriately. Our data indicate comparable TENT assessment data from examiners with or without formal postgraduate neuropsychology training. This finding is of practical benefit for clinical and research teams. In our own project we trained clinical trials assistants to run assessments, adding variety to their work, and expanding the range of team members who can carry out assessments. All examiners received standardized training and participate in regular team meetings to discuss assessment scenarios that arise, fostering a consistent approach through experience sharing.

### Benefits of TENT

The potential benefits of TENT are substantial in both research and clinical settings. The benefits of teleneuropsychology in general are already well understood, and these benefits extend to TENT. TENT enables remote assessment facilitating a broad geographic reach, along with other benefits that come from this (i.e., increased access to services and research participation for a broad range of individuals, convenience, reduced time and financial burden, ease of follow-up/continuity of care). In so doing, it also allows for centralized clinical teams and research trial designs, simultaneously improving the ability to standardize and harmonize within teams and reducing the costs of service delivery/research. This in turn has important implications for scalability, a crucial requirement in the move towards population-level health care and research [68].

TENT has additional benefits beyond the benefits of teleneuropsychology in general. Being examiner-led, TENT preserves human-to-human interaction, which is important for maintaining participant engagement and data quality [3], something that is lost in *unsupervised computer-based cognitive testing*. However, incorporating computer-assisted testing also confers benefits: the use of on-screen examiner instructions should improve the standardization of assessments; stimulus delivery and response timing can be precisely controlled and monitored; data/responses are readily captured at the individual item level; scoring (raw and normative) occur automatically in real time, minimizing scope for human error and improving efficiency; and data records are intrinsically electronic and readily ingestible into central databases. Further, many of the features of TENT (e.g., automated stimulus delivery, automatic scoring/data recording, interfaces for efficient response recording, quick navigation between tasks) likely increase the overall efficiency of assessments compared to using traditional analog measures, reducing the burden on participants and assessors. Capturing item-level responses also makes analyses such as those informed by item response theory readily accessible, which can facilitate the refinement and shortening of measures while maintaining sound psychometric properties. These are benefits not realized by simply ‘shoehorning’ existing practices into a videoconferencing medium without capitalizing on the use of technology. Incorporating screen-sharing means that ‘off the shelf’ test materials can also be used where desired, enabling flexibility of testing beyond specific tasks implemented in TENT. Finally, we note that the benefits of TENT are not restricted just to remote telehealth. The software can just as readily be applied with many of the above benefits still conferred, to in-person assessments with the participant on one device and the examiner on a second.

### Future directions

We plan to continue to expand the TENT software. While TENT has been used here with working-age adults with seizure disorders, it will have utility for individuals with other clinical conditions (e.g., acquired brain injury, neurodegenerative disorders) and across a broader age range, from paediatrics through to older adults. Research indicates that remote neuropsychological assessment is feasible and yields comparable results to in-person assessment across a range of ages and conditions [e.g., 69–71]. Of note, within our cohort, we have successfully used the software with people with mild intellectual disability demonstrating the feasibility of the software for use in impaired populations. Expanding the use of TENT to other populations will benefit from broadening the range of tasks available and extending the age range of our normative data. For instance, we did not include a measure of visuospatial functioning as this domain is rarely impacted in epilepsy, though it will be advantageous to sample this in other contexts. Copying tasks can be easily administered using the screen-share feature, as has been validated in the literature [67, 69]. We are actively exploring the addition of videoconference-native visuospatial tasks. Further, the data presented here touches primarily upon gross ‘aggregate’ measures (e.g., total number of words recalled across learning trials). However, as alluded to above, there is considerable scope to evaluate item-level data for specific patterns that map to specific clinical features, and to refine measures to make them more efficient with minimal trade-off in accuracy/precision [72]. We are also conducting latent variable analyses of the data collected via TENT to identify the underlying structure of the data. This will enable extraction of summary indices that are more reliable than the individual tasks themselves, and that can be used in data reporting and linkage to multimodal analyses. Importantly, we are also currently collecting test–retest data for TENT, to determine instrument reliability and change indices [73].

## Conclusion

We have developed software for providing examiner-administered, computer-assisted cognitive testing, and validated it within a large cohort of healthy volunteers and individuals with DRE. The TENT software provides for human-led cognitive assessments that exploit the benefits of technology-assisted testing and can be used for remote assessment. This approach draws upon the strengths of the traditional assessment model while modernizing contemporary neuropsychological practice.

## Supplementary Information

Below is the link to the electronic supplementary material.

Supplementary file1 (DOCX 27 KB)

## Data Availability

The datasets used and/or analyzed during the current study are available from the corresponding author on reasonable request.

## Abbreviations

AEP: : Australian Epilepsy Project
ASM: : Antiseizure medication
ATL: : Anterior temporal lobectomy
BF: : Bayes factor
BNT: : Boston Naming Test
DRE: : Drug-resistant focal epilepsy
FSIQ-II: : Full-Scale Intelligence Quotient-II
FUS: : First unprovoked seizure
HS: : Hippocampal sclerosis
ICC: : Intra-class correlation coefficient
NDE: : Newly diagnosed epilepsy
PCA: : Principal components analysis
RAVLT: : Rey Auditory Verbal Learning Test
RCF: : Rey Complex Figure Test
RT: : Reaction time
SDMT: : Symbol Digital Modalities Task
TENT: : Telehealth Enabled Neuropsychological Testing
TMT: : Trail Making Test
WASI-II: : Weschler abbreviated scale of intelligence–second edition

## Footnotes

1 Indeed, the attempt to reproduce the in-person experience has led some to post stimulus materials and record forms to participants in advance, which then must be mailed back to the examiner.
