# Supplementary material for: Telehealth enabled neuropsychological testing (TENT): a new platform for examiner-led, digital cognitive assessment
Source: J Neurol. 2026 Mar 9;273(3):192. doi: 10.1007/s00415-026-13732-1 (PMC12971734; doi:10.1007/s00415-026-13732-1)
Supplement: Supplementary file 1 — Supplementary file1 (DOCX 27 KB) [file 415_2026_13732_MOESM1_ESM.docx]

## Supplementary Information: Detailed Description of TENT Tasks

### Category Fluency

Category Fluency is a measure of semantic knowledge and lexical retrieval. Participants are asked to name as many different types of animals as they can within 60 seconds. The examiner classifies each participant response as *valid* or *invalid* (e.g., repetitions, out of category responses). Administration time is approximately two minutes. The primary measure of interest is the total number of unique animal names provided.

### Confrontation Naming

Confrontation Naming assesses visual naming ability. Participants name 60 photographed objects as quickly as possible. The task includes items that overlap with existing tasks (e.g., Boston Naming Test [1], Sydney Language Battery [2], Multilingual Naming Test [3]) and novel items. Examiners record responses in real-time, noting correct answers, errors, and word-finding difficulties. Semantic cues are provided only for misperceived stimuli. If no response is given within 5 seconds, a prompt is provided ("Do you think you would know it if you heard it?"). Each item has a 20-second limit. Multiple attempts are recorded, with the final response counted. Administration time is about 5.5 minutes, with the primary measure being the number of correctly named items.

## Digit Span

Digit Span assesses immediate attention and working memory. The examiner reads predefined number sequences of increasing length at a rate of one digit per second. Participants repeat these forwards (Forward Span) or backwards (Backward Span). The examiner records, via their keyboard, participant responses verbatim which are then automatically scored. The task is discontinued after two incorrect responses at a given span length. Forward span is tested first, then backward span. Administration time is about 4 minutes. Primary measures are the maximum forward and backward spans achieved.

### Figural Learning and Figural Recognition

Figural Learning and Figural Recognition are measures of non-verbal learning and delayed recognition, respectively. All stimuli are presented on, and testing occurs on, a grey triangular lattice. The eight stimuli are figures made by thickening and blackening five contiguous lines on the lattice. The figures are presented one at a time for 2.8s each, with a 0.2s interstimulus interval. Participants are then shown a blank lattice and asked to reproduce as many figures as they can by selecting lines (the lines being thickened and blackened once selected). Participants complete three learning trials.

Recognition is tested after approximately 20 minutes using a four-alternative forced-choice procedure, testing separately for figure shape (i.e., the five line configuration) and then location (i.e., it’s location on the underlying lattice). Administration time for Figural Learning is approximately 7.5 minutes, while administration time for Figural Recognition is approximately 2 minutes. Key measures are total items recalled across the learning trials (Figural Learning) and total correct configuration and position recognition (Figural Recognition).

### Finger Tapping

Finger Tapping measures manual repetitive motor speed. Participants repeatedly press the ‘B’ key on their keyboard using their right (two trials) and left (two trials) index finger as quickly as they can for 10 seconds. They are first shown a demonstration video, illustrating the desired tapping technique, with the heal of the hand resting on a surface and the index finger moving in isolation at the metacarpophalangeal joint. The participant’s self-reported hand dominance is recorded. Administration time is approximately 2 minutes. The primary measures of interest are the mean tap count for the dominant and non-dominant hands.

### Irregular Word Reading

Irregular Word Reading estimates premorbid intellectual ability based upon the pronunciation of words with irregular grapheme-to-phoneme conversion rules (e.g., “debt”). The task is akin to other irregular word reading measures such as the Wechsler Test of Adult Reading [4], the Test of Premorbid Function [5], and the National Adult Reading Test [6], exhibiting some overlap with these materials. The task contains 50 irregular words ordered according to their difficulty (based on pilot work). Words are shown one at a time in lower case font on the participant’s screen and the participant asked to say the word aloud. The examiner records, for each word, whether it was pronounced correctly or not. Administration of the task is discontinued if the participant makes eight consecutive errors. Administration time is approximately 4 minutes. The primary measure is the total number of correctly pronounced words.

### Letter Fluency

Letter Fluency is a measure of executive function and lexical retrieval. Participants are asked to produce as many different words as they can beginning with a particular letter within 60 seconds. Participants are instructed not to use proper nouns (words they would normally capitalise), and not to repeat a word and simply add an ending (e.g. -ed, -s, -ing). Three letters are tested across separate trials (B, H, R). The examiner records each response as either *valid* or *invalid* (e.g., repetitions, incorrect initial letter, proper nouns). Administration time is approximately 5 minutes. The primary measure is the total number of unique words provided, summed across the three trials.

### Reaction Time

Reaction Time measures how quickly a motor response can be generated upon presentation of a visual stimulus (i.e., simple reaction time). On each trial, participants are shown a large green circle at the centre of their screen and are required to respond as quickly as possible by pressing the ‘B’ key. Interstimulus intervals are drawn randomly from a uniform distribution in the range 1-2 seconds. Responses shorter than 100ms are deemed biologically implausible and therefore invalid. Trials continue to be shown until 30 valid responses have been collected. Administration time is approximately 2 minutes. The primary measure of interest is the median reaction time over the last 25 valid trials.

### Spatial Stroop

Spatial Stroop measures attentional control and choice reaction time. It is adapted from a task described in Baldo et al [7]. Participants respond, using designated keys for their left and right index fingers, to the words LEFT or RIGHT shown successively on screen in random order. In the *Congruent* condition, they respond with their left index finger for LEFT and their right index finger for RIGHT. Incorrect responses turn the stimulus red, which remains on screen until a correct response is provided. Trials are separated by a 500ms inter stimulus interval. After 50 trials, the mapping is reversed (e.g., right hand required for LEFT) for 50 trials in an *Incongruent* condition. Administration time is approximately 4.5 minutes. Primary measures are median reaction times and error counts for both conditions.

### Symbol Decoding

Symbol Decoding measures attention and processing speed; its implementation in TENT has been described in Chapman et al [8]. Participants are shown a key pairing nine unique symbols with the numbers one though nine. The participant must orally decode subsequent rows of symbols in line with the provided key, by telling the examiner the number associated with each symbol. Participants practice 10 symbols to demonstrate their understanding of the task. During the test phase participants see the key at the top of the screen, underneath which are four rows each containing ten symbols. Participants are instructed to complete as many items as they can within 90s, working from left to right across each row. The Examiner screen displays the same stimulus set viewed by the participant, but with the correct responses included in the box beneath each symbol (not seen on the participant screen). The examiner records whether each response was correct (by clicking the number) or incorrect (by clicking the symbol) and recorded responses can be amended in real time by the examiner (e.g., if the participant subsequently self-corrects an error). When the examiner records the final response on a given page the screen automatically advances to a new page. Participants are instructed to use their cursor or finger to track their progress if desired. Administration time is approximately 3.5 minutes. The primary measure of interest is the number symbols correctly decoded within 90s.

### Trail Making

Trail Making measures attention, processing speed and mental flexibility; its implementation in TENT has been described in Chapman et al [8]. Participants are shown 25 circles distributed across their screen, containing either numbers (Part A: numbers 1-25) or numbers and letters (Part B: numbers 1-13, letters A-L). Participants are required to click on the numbers in ascending sequence (Part A) or the numbers and letters in alternating ascending sequence (Part B). Each trial (Part A, Part B) is preceded by a practice trial to ensure task understanding. As participants respond, each correctly selected number/letter turns grey and is joined to the preceding number/letter with a black line to create a visual ‘trail’. If a participant makes an error (i.e., clicks on an out of sequence circle), the incorrectly selected number/letter turns red, the entire screen pulses a dim red, and a written message appears at the top of the screen directing the participant back to their last correct response. The error message provides increasing guidance if successive errors are made. This method of handling errors is demonstrated during the explanation of the task. During the task the examiner sees the participant’s display with the location of the participant’s cursor mirrored in real time. The examiner provides additional verbal or visual (cursor) prompts if the participant does not recognise errors and/or could not be successfully redirected via the provided prompts. Administration time is approximately 4 minutes. The primary measures of interest are the completion times on the Part A and Part B trials.

### Word List Learning and Word List Delay

Word List Learning and Delay assess verbal anterograde memory. The task is akin to an abbreviated form of the Rey Auditory Verbal Learning Test (RAVLT; [9–11]). Participants are presented with a list of 15 unrelated words, spoken aloud by the examiner at a rate of one word every 1.2 seconds as per a timing pulse displayed on the examiner’s screen. The task uses a novel 15-item word list, distinct from commonly used variants of the RAVLT. Participants are then asked to recall as many words as they can from the list, in any order. The examiner records each word correctly recalled, plus any intrusions and repetitions. This process is repeated for a total of three learning trials. For Word List Delay participants are asked, approximately 15 minutes after the conclusion of the last learning trial and without forewarning, to recall as many words as they can. The administration time for *Word List Learning* is approximately 3.5 minutes, and for *Word List Delay* is approximately 1 minute. The primary measures of interest are *Total Learning Score* (sum of total words recalled across Trials 1–3), *Delay Score* (total words recalled on the delay trial) and *Loss Score* (Trial 3 score minus delay score). We also calculated a *Proportional Loss* across delay score (loss score divided by Trial 3 score).

### Non-Word Rhyming

TENT contains two tasks used to train participants for in-scanner cognitive paradigms collected as part of their participation in the Australian Epilepsy Project. One of these is *Non-Word Rhyming* based on that described in Tailby et al [12]. In brief, this task measures grapheme-to-phoneme conversion. Participants are asked to judge, as recorded via button press, whether two visually presented non-words rhyme (e.g. “flig” and “brank”). Blocks of rhyming judgements (20 judgements in total) are alternated with blocks of visuospatial pattern judgements (20 in total), where participants are asked to judge whether two visually presented patterns of forward and backslashes are identical (e.g., “/\///” and “//\//”). Each block comprised 4 stimulus pairs, with each stimulus pair shown for 4 seconds with a 500ms interstimulus interval. Administration time for *Non-Word Rhyming* is approximately 5.5 minutes. The primary measures are proportion correct and median reaction time in the non-word rhyming and pattern matching conditions.

### Spatial n-Back

The second task used in conjunction the AEP scanning protocol is a *Spatial n-Back* task based on the paradigm described in Callicott et al [13]. *Spatial n-Back* measures visuospatial working memory and choice reaction times. Participants are presented with yellow dots shown one at a time (for 0.75s followed by a 1.25s interstimulus interval) at one of four possible locations on the screen, positioned at the vertices of a virtual diamond centred on the midpoint of the display. In the 0-back condition, participants indicate the current dot's location by pressing one of four pre-assigned keys on their keyboard. In the 1-back condition, they indicate the previous dot's location. The task alternates between 0-back and 1-back blocks, with a sequence of 11 dots per block. The primary measures are proportion correct and median reaction time in the 0-back and 1-back conditions.

## References

1. Kaplan E, Goodglass H, Weintraub S (1983) The Boston Naming Test. Lea & Febiger. Philadelphia, PA

2. Savage S, Hsieh S, Leslie F, et al (2013) Distinguishing subtypes in primary progressive aphasia: application of the Sydney language battery. Dementia and geriatric cognitive disorders 35:208–218

3. Gollan TH, Weissberger GH, Runnqvist E, et al (2012) Self-ratings of spoken language dominance: A Multilingual Naming Test (MINT) and preliminary norms for young and aging Spanish–English bilinguals. Bilingualism: language and cognition 15:594–615

4. Wechsler D (2001) Wechsler Test of Adult Reading: WTAR. Psychological Corporation

5. Wechsler D (2009) Test of premorbid functioning. San Antonio, TX: The Psychological Corporation

6. Nelson HE, Willison J (1991) National adult reading test (NART). Nfer-Nelson Windsor

7. Baldo JV, Shimamura AP, Prinzmetal W (1998) Mapping symbols to response modalities: Interference effects on Stroop-like tasks. Perception & Psychophysics 60:427–437. https://doi.org/10.3758/BF03206864

8. Chapman JE, Helmstaedter C, Abbott DF, et al (2025) Videoconference-integrated, computer-assisted cognitive testing improves the remote assessment of processing speed and attention. Journal of the International Neuropsychological Society 1–10

9. Rey A (1941) L’examen psychologique dans les cas d’encéphalopathie traumatique.(Les problems.). Archives de psychologie

10. Schmidt M (1996) Rey auditory verbal learning test: A handbook. Western Psychological Services Los Angeles, CA

11. LaForte EM, Hook JN, Giella AK (2023) National Institutes of Health (NIH) Toolbox® V3 Technical Manual

12. Tailby C, Abbott DF, Jackson GD (2017) The diminishing dominance of the dominant hemisphere: Language fMRI in focal epilepsy. NeuroImage: Clinical

13. Callicott JH, Mattay VS, Bertolino A, et al (1999) Physiological characteristics of capacity constraints in working memory as revealed by functional MRI. Cerebral cortex 9:20–26
